# Cryoprotective Roles of Carboxymethyl Chitosan during the Frozen Storage of Surimi: Protein Structures, Gel Behaviors and Edible Qualities

**DOI:** 10.3390/foods11030356

**Published:** 2022-01-26

**Authors:** Xiangwei Zhu, Minglang Zhu, Diheng He, Xueyin Li, Liu Shi, Lan Wang, Jianteng Xu, Yi Zheng, Tao Yin

**Affiliations:** 1 National “111” Center for Cellular Regulation and Molecular Pharmaceutics, Key Laboratory of Fermentation Engineering (Ministry of Education), Hubei Key Laboratory of Industrial Microbiology, Hubei University of Technology, Wuhan 430068, China; datouzhu1216@163.com (M.Z.); hdiheng@163.com (D.H.); xiali12282021@163.com (X.L.); 2 Institute for Farm Products Processing and Nuclear-Agricultural Technology, Hubei Academy of Agricultural Science, Wuhan 430064, China; shiliu_hzau@163.com (L.S.); lilywang_2016@163.com (L.W.); 3 Department of Grain Science and Industry, Kansas State University, Manhattan, KS 66506, USA; jianteng@ksu.edu (J.X.); yzheng@ksu.edu (Y.Z.); 4 College of Food Science and Technology, Huazhong Agricultural University, Wuhan 430070, China

**Keywords:** carboxymethyl chitosan, frozen surimi, myofibrillar protein, denaturation, gelling properties

## Abstract

Carboxymethyl chitosan (CMCh) is an ampholytic chitosan derivative that manifests versatile applications in food industry, such as antibacterial ingredients and nutritional additives. However, its use as a cryoprotectant remains under-researched. In this study, the cryoprotective effect of CMCh oligosaccharide (CMCO) on frozen surimi (silver carp) was systematically investigated in terms of protein structures, gelling behaviors, and sensory qualities. CMCO (0.6%) was incorporated in the surimi before frozen storage (−18 °C for 60 days) while the commercial cryoprotectant (4% sucrose, 4% sorbitol) was used as a positive control. Results indicated that CMCO could inhibit the freezing-induced denaturation of myofibrillar protein, whose values of solubility, Ca^2+^-ATPase and sulfhydryl content were 24.8%, 64.7%, and 17.1% higher than the nonprotected sample, respectively, while the surface hydrophobicity was 21.6% lower. Accordingly, CMCO stabilized microstructure of the surimi gels associated with improved gel strength, viscoelasticity, water-holding capacities, and whiteness. Moreover, the cryoprotective effect of CMCO with higher degree of carboxymethyl substitution (DS: 1.2) was more pronounced than that of low-DS-CMCO (DS: 0.8). Frozen surimi treated with high-DS-CMCO achieved competitive gelling properties and sensory acceptability to those with the commercial counterpart. This study provided scientific insights into the development of ampholytic oligosaccharides as food cryoprotectants.

## 1. Introduction

Surimi is a reconstituted food used for the production of various seafood dishes, such as fish balls, cakes, and croquettes [1,2]. Among all ingredients of the fish muscle, myofibrillar protein (MP), a salt-soluble protein, is mainly responsible for surimi functionalities [3]. Like other uncured meats, the shelf life of surimi is largely depending on frozen storage [3,4,5], which restrains intrinsic enzyme activities, microbial growth, and lipid oxidation of surimi during long-term preservation [6,7]. However, undesirable quality deteriorations still occur (e.g., water loss, structural weakening, and nutrition decay), and most of them are related to MP denaturation [8], which owes to the effect of ice crystallization, protein concentration and oxidation [9].

To address those problems, appropriate uses of food-grade cryoprotectants are necessary [10]. Given the unexpected taste or high caloric values of commercial cryoprotectants, such as sugars, polyols, and phosphates [11], research interest shifted to other natural additives derived from food proteins and saccharides. Among which, oligosaccharides, i.e., carrageenan oligomer [12], cellobiose [13], and konjac oligo-glucomannan [14] etc., have drawn particular attentions owing to their pronounced health benefits and consumer acceptability [15].

Oligosaccharides function as cryoprotectants through: (1) inhibiting ice recrystallization in frozen surimi and reducing physical damages to MP structures [9], (2) forming saccharide-MP binary complex to prevent MP from freezing-induced aggregation [16]. It is noteworthy that both ice-inhibition and complexation effects of saccharides were dependent on their strong polarities [9,17]. Therefore, some intrinsic structural characteristics, such as the charge properties, of exogenous oligosaccharides are believed to govern their cryoprotective effects. For example, Zhang et al. [18,19] reported that alginate oligosaccharide and trehalose could prevent the freezing denaturation of myosin. Though both saccharides exhibited the same degree of polymerization (DP = 2n), the charged alginate oligomer had stronger protecting effect than the noncharged trehalose.

Chitosan is an acid-soluble cationic polysaccharide that has been widely used as food additives [20,21]. To improve its solubility in water, carboxymethylation is mostly carried out [22]. The derived carboxymethyl chitosan (CMCh, Figure S1) is a typical ampholytic polysaccharide [23,24]. It demonstrates outstanding moisture-retention capacity [22], which is the key to regulate the water state and ice crystallization at subzero temperatures. Furthermore, the ampholytic structure of saccharide was also reported to be effective in modulating the protein aggregation [25]. On the basis of these observations, the ampholytic CMCh was a potential cryoprotectant to proteins. However, the development of CMCh-based additives remained less in frozen food when compared with other charged or neutral saccharides, such as chitosan (+), carrageenan (−), and konjac glucomannan (non-ionic) [26,27]. Thus, the objective of this study is to: (1) investigate how the ampholytic structure of CMCh influences its cryoprotective effect to frozen surimi proteins, and (2) evaluate the edible properties of obtained surimi gels.

Since the functional properties of charged saccharides are highly related to their charge densities [25,26], CMCh with different degrees of carboxymethyl substitution (DS) were selected as cryoprotectants in this study, which were hydrolyzed to oligosaccharides and added to surimi before freezing. The storage stability of MP was thoroughly characterized, while the gelling behaviors and edible qualities of frozen surimi were also evaluated, including the rheological properties, microstructures, mechanical strength, water-holding capacities, sensory scores, and whiteness. This work aims at a paradigm shift for the development of ampholytic oligosaccharides as high-performance cryoprotectants for aquatic food preservations.

## 2. Materials and Methods

### 2.1. Materials

Alive farm raised-silver carp (Hypophthalmichthys molitrix, weighing 2.0 ± 0.1 kg) were purchased from a local market (Wuhan, China), transported to laboratory within 15 min, and subjected to percussive stunning. Carboxymethyl chitosan with two different DS, CMCh-A and -B, were kindly donated by Haobo biotech Corp (Henan, China). The DS values of CMCh-A and -B were characterized to be 0.8 and 1.2 by using the potentiometric titration [28]. Both CMCh exhibited similar molecular weight (M_w_) at about 6.02 × 10^4^ Da. Chitosanase, with an activity unit at 200,000 U/g, was purchased from Shengda biotech Corp (Henan, China). All other chemicals were purchased from Aladdin Bio-chem Technology Co., LTD (Shanghai, China). Ultrapure water was used in all experiments unless specified otherwise.

### 2.2. Preparation of CMCh Oligosaccharide

The CMCh oligosaccharide (CMCO) was prepared according to previous studies with some modifications [29,30]. Briefly, 500 mL of CMCh solution (10 mg/mL) was prepared at pH 5, and an aliquot of 60 mg chitosanase was added into the solution. The hydrolysis reaction was carried out at 50 °C for 5 h and terminated by heating the mixture at 100 °C for 10 min. The obtained solution was neutralized, filtered, and concentrated with a vacuum rotary evaporator (IkA-Works Inc., Staufen, Germany). Then, the hydrolysate was precipitated by mixing with 10 volumes of ethanol. After being redissolved in the water, the solution was filtered through a polymer membrane (Mw cut-off: 10 KDa) and the filtrates were lyophilized. The average DP for both CMCO-A and CMCO-B was characterized to be approximately 6.8, as reflected by the contents of reducing sugars.

### 2.3. Preparation of Frozen Surimi

The frozen surimi was prepared according to Zhangt et al. [31]. The fish was killed and its head, scales and internal organs were removed. Fresh fish meat was cut and washed three times by five volumes of distilled ice water. Then, the surimi was dispersed in 0.5% NaCl solution at the ratio of 1:2 (*w*/*v*) and dehydrated with nylon net to reach a final moisture content at 78%. The protein content in surimi was determined to be 15.2% through the Kjeldahl method. The obtained surimi was added with CMCO-A (0.6%), CMCO-B (0.6%) or commercial cryoprotectant (4% sucrose and 4% sorbitol), respectively, and packaged in polyethylene bags with the size of 20 × 15 × 2 cm^3^. Surimi without any cryoprotectant added was set as a blank control. All samples were frozen at −18 °C in air (with a freezing rate of 1.2 °C/min) and conditioned overnight to achieve uniform initial temperatures. Then, the surimi was transferred to a freezer and stored at −18 °C for 0, 10, 20, 30, 45 and 60 days. The frozen surimi was thawed at 4 °C for 20 h before further analysis.

### 2.4. Characterizations of MP in Frozen Surimi

#### 2.4.1. Content of Salt-Soluble Protein

MP of the surimi was prepared by following the processes of Lin et al. with modifications [7]. Frozen surimi (5 g) was mixed with 45 mL of 0.6 M KCl solution (pH 7.0) and homogenized at 9000 rpm for 100 s (an operation of 20 s with an interval of 15 s). The homogenate was conditioned in an ice water bath for 30 s and centrifuged at 9000 rpm, 4 °C for 20 min. The supernatant was collected and mixed with three volumes of cold deionized water followed by centrifugation at 6000 rpm, 4 °C for 20 min. The precipitate was re-suspended in 20 mL of 0.6 M KCl solution and centrifuged again at 6000 rpm, 4 °C for 20 min. Finally, content of salt-soluble protein in the supernatant was determined by the Biuret method.

#### 2.4.2. Ca^2+^-ATPase Activity

Ca^2+^-ATPase activity was determined according to Liu et al. [14]. Briefly, the Ca^2+^-ATPase assay was performed at 37 °C for 10 min in a reaction medium that consisted of 0.5 M KCl, 5 mM CaCl_2_, 25 mM Tris-maleate (pH 7.0), 6 mg/mL MP, and 1 mM ATP. The reaction was terminated by adding HClO_4_ at 5% final concentration. The mixture was centrifuged at 9000 rpm for 10 min, and the released inorganic phosphate in the supernatant was measured by using phosphomolybdate method. The activity of Ca^2+^-ATPase of MP was expressed as Pi amount per mg of MP per reaction time liberation min^−1^ (μmol/mg/min)^−1^.

#### 2.4.3. Surface Hydrophobicity

Surface hydrophobicity of MP from the frozen surimi was characterized by using 8-anilino-1-naphthalenesulfonic acid (ANS) as the fluorescence probe according to Lin et al. with modifications [7]. The MP solution was diluted with 0.6 M KCl-10 mM phosphate buffer (pH 6.0) to achieve a series of protein concentrations of 0.2, 0.3, 0.5 and 1.0 mg/mL, and 20 μL of 8 mM ANS-0.1 M phosphate buffer (pH 7.0) was added to 1 mL of the prepared protein solution. Fluorescence intensity (FI) of the mixture was measured immediately by using a fluorophotometer (F-4600, Hitiachi High Technologies Co., Tokyo, Japan) with excitation wavelength at 390 nm and emission wavelength at 420 nm. The recorded FI vs. the concentration of MP (mg/mL) was plotted, and the initial slope was used as the index of protein surface hydrophobicity.

#### 2.4.4. Concentration of Sulfhydryl Group

The concentration of sulfhydryl (–SH) group was determined via DTNB (5,5′-Dithiobis-2-nitrobenzoic acid) assay as described by Jiang [32]. In brief, 1 mL of MP solution (4 mg/mL) was mixed with 9 mL buffer A (8 mol/L urea, 2% SDS and 10 mmol/L EDTA at pH 8.0), and 4 mL of the mixture was mixed with 0.5 mL buffer B (10 mM DTNB and 0.2 M Tris-HCl, pH 8.0). After incubation at 40 °C for 25 min, absorbance of the solution was determined at 412 nm. The concentration of –SH group was calculated as Equation (1):

(1) $$x=\frac{A\;\times \;D}{C\;\times \;B}$$

where *x* represents the SH content (mol/g); D is the dilution factor; A is the absorbance of the mixture solution; C is the molar extinction (13,600 mol^−1^·cm^−1^·L); B is the protein concentration (mg/mL).

#### 2.4.5. Intrinsic Fluorescence Intensity

Fluorescence intensity (FI) of MPs was measured with a fluorescence spectrophotometer (F-4600, Hitiachi High Technologies Co.) according to the method described by Walayat et al. [33]. Previously prepared MP solution (Section 2.3) was diluted to 0.05 mg/mL by using 0.6 M NaCl solution. The scan wavelength was in the range of 300 to 450 nm, with the scanning speed at 1000 nm/min, and the excitation wavelength was 295 nm with both excitation and emission slits at 10 nm.

### 2.5. Preparation of Surimi Gels

Surimi gel was prepared according to the method described by Tao et al. [8]. Frozen surimi was thawed at 4 °C, chopped for 5 min, mixed with 2.5% NaCl addition followed by chopping for another 5 min. The obtained surimi sol was then stuffed into a plastic polyvinylidene case and conditioned at 40 °C for 60 min followed by heating at 90 °C for 30 min (Figure S2). The obtained heat-set surimi gels were cooled in ice water for 30 min and stored at 4 °C overnight before further analysis.

### 2.6. Characterizations of Surimi Gels

#### 2.6.1. Scanning Electron Microscopy

Scanning electron microscopy (SEM) was used to evaluate the microstructures of surimi gels. A cubic Surimi gel (3 × 3 × 3 mm^3^) was conditioned in 2% glutaraldehyde at 4 °C for 18 h and dehydrated with a series of concentrations of ethanol (50, 70, 80, 90, and 100%), followed by lyophilization. Then, gel samples were mounted on an aluminum stub, sputter coated with gold, and observed by SEM (Hitachi S–3500 N, Hitachi, Japan) at an accelerating voltage of 10 kV.

#### 2.6.2. Rheology Test

Small amplitude oscillatory strain (SAOS) tests were performed with a Physica controlled stress rheometer (MCR-301, Anton Paar, Graz, Austria). A small piece of surimi gel was loaded between two parallel plates (20 mm diameter and 1 mm gap). Disk-shaped samples were mounted on the lower plate of the rheometer at 20.0 ± 0.1 °C and allowed to rest for 15 min before analyses. Then, a frequency sweep test (0.1–100 Hz) was carried out to evaluate the rheological variations of the gels prepared from surimi stored for different periods of time. The strain was fixed at 0.5% which was within the viscoelastic region (LVR), and the storage modulus (G′) and loss modulus (G′′) were recorded. Values of tan δ were expressed as G′′/G′ at the testing frequency of 1 Hz and the temperature of 20 °C.

#### 2.6.3. Gel Strength

Gel strength was characterized on a TA. XT. plus Texture analyzer (SMS, Surrey, UK) according to the method of Liu et al. [14]. Surimi gels were cut into 25 mm high cylindrical shape slices. A slice was horizontally placed on the platform and penetrated by a spherical probe (type P/0.25). The testing speed of probe was 1 mm/s, the trigger point load was 0.1 N, and the depth of probe penetration was 10 mm. Gel strength (g/cm) was calculated by multiplying the breaking force (g) by penetration distance (mm).

#### 2.6.4. Water-Holding Capacity (WHC)

WHC of surimi gels was measured according to the method described by Li et al. with minor modifications [4]. Surimi gels were cut into the thickness of 2.5 mm and centrifuged for 10 min at 1000 g and 4 °C, and the released moisture was absorbed by filter paper. The weight of the gels was recorded before (W1) and after centrifugation (W2). WHC was calculated as Equation (2):

WHC (%) = (W1 − W2)/W1 × 100%(2)

#### 2.6.5. Whiteness

The whiteness of surimi gel was determined with a colorimeter (CR-400, Konica Minolta, Osaka, Japan) by measuring L* (lightness), a* (redness/greenness) and b* (yellowness/blueness) values. Whiteness was calculated as Equation (3):

Whiteness = 100 − 100 − L*)^2^ + a*^2^ + b*^2^] ^1/2^ (3)

#### 2.6.6. Sensory Assessment of Surimi Gels

Surimi gels (prepared in Section 2.5) were cut into square pieces (1 × 1 × 2 cm) for sensory assessment. The evaluation panelist was composed of 20 trained individuals, and the whole evaluation process was performed in a professional sensory evaluation laboratory at room temperature. A five-point hedonic score was recorded for each surimi gel, ranging from 5 (extremely like) to 1 (extremely dislike) in terms of the taste, smell, juiciness, color, texture and overall acceptability of the cooked surimi gel.

### 2.7. Statistical Analysis

All characterizations were repeated three times, and the reported results were shown as the mean ± standard deviation. Significant difference among treatments was analyzed by using SPSS (Version 13.0 SPSS, Chicago, IL, USA) through analysis of variance (ANOVA) with Duncan’s test. Statistical significance was accepted at *p* < 0.05.

## 3. Results and Discussions

### 3.1. Effect of CMCO on Storage Stability of MP

#### 3.1.1. Salt-Soluble Protein Content

**Figure 1:**
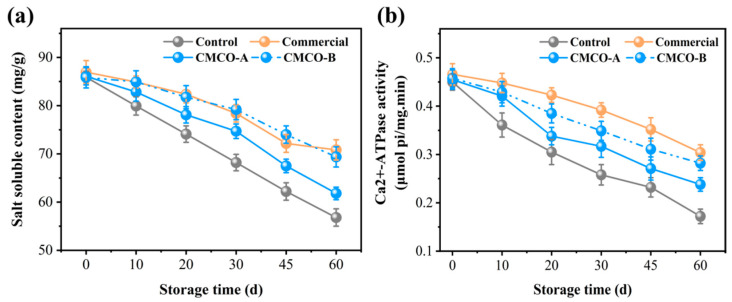
Effects of CMCO on salt-soluble protein content (**a**) and Ca^2+^-ATPase activity (**b**) of MP at different storage time.

MP is the dominant salt-soluble protein in the surimi, whose solubility variation is an important indicator to reflect its denaturation during frozen storage [34]. As shown in Figure 1a, the salt solubility of MP from all groups of surimi were about 86 mg/g before freezing and kept decreasing during the entire storage period. After 60 days, the solubility of the control group reached 58 mg/g. The decrease of protein solubility could be attributed to the freezing-induced denaturation/aggregation of MP in surimi as the storage time extended [12,35]. With the addition of cryoprotectants, the MP solubility of frozen surimi was significantly higher, whose values were 62, 70, and 72 mg/g, respectively, regarding to CMCO-A, -B and commercial group. Similar to most other saccharide cryoprotectants, CMCO could restrain the movement of water molecules and inhibit the growth of ice in frozen surimi, which alleviated the MP denaturation. Moreover, the MP solubility of CMCO-B group was higher than that of CMCO-A at the same storage time. It has been shown that the complexity of hydrogen bonding enhanced in the food matrix as the DS of charged polysaccharide increased [20,36]; thus, water molecules became more bounded to the protein networks, which led to a better cryoprotective effect to the MP, i.e., CMCO-B group > CMCO-A group, whose content of salt-soluble protein was 70 and 62 mg/g, respectively.

#### 3.1.2. Ca^2+^-ATPase Activity

Ca^2+^-ATPase activity is an important indicator to measure the structural integrity of MP in fish muscle [8]. The protein conformations can be changed along with the breakdown of intermolecular noncovalent bonding during storage, resulting in the decrease of Ca^2+^-ATPase activity [35]. Figure 1b showed that the initial Ca^2+^-ATPase activity of MP of all frozen surimi samples were about at 0.45 μmol pi/mg.min before freezing and decreased to different degrees as storage time extended. The Ca^2+^-ATPase activity of the control group experienced the most significant decrease (~60%) to 0.17 μmol pi/mg.min at day 60. In consistent with the results of MP solubility (Section 3.1), the use of cryoprotectants (i.e., CMCO-A/-B and the commercial additive) inhibited the protein denaturation. Specifically, the values of Ca^2+^-ATPase activity of the CMCO-A (0.23 μmol pi/mg.min) and -B (0.28 μmol pi/mg.min) were significantly higher than that of the control group (0.17 μmol pi/mg.min) at day 60, even though they also decreased by 44.4% and 33.3%, respectively, during storage. Meanwhile, the CMCO-B group achieved comparable 60-day Ca^2+^-ATPase activity (i.e., 0.28 vs. 0.30 μmol pi/mg.min) to the commercial group. A similar cryoprotective effect of pullulan was also reported by Jiang et al. [37].

#### 3.1.3. Surface Hydrophobicity and Sulfhydryl Content

**Figure 2:**
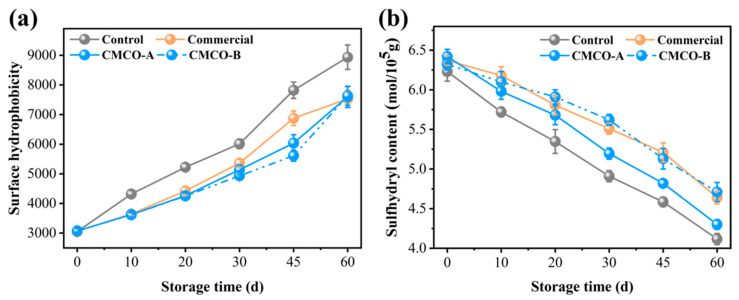
Effects of CMCO on surface hydrophobicity (**a**) and sulfhydryl content (**b**) of MP at different storage time.

Variations of surface hydrophobicity reflect conformational changes of MP at different physical and chemical states. A reduced surface hydrophobicity suggests that the protein was less denatured (unfolded), and less hydrophobic groups were exposed, thus limiting the protein binding with the fluorescence probe [7]. As depicted in Figure 2a, surface hydrophobicity of the control group increased significantly from 3000 to 8800 after 60 days of storage. This phenomenon was reasonably due to the freezing-induced MP denaturation. Ice crystals that formed in surimi destroyed the hydration layer around the peptides, which resulted in more exposed hydrophobic amino acid residues and higher protein surface hydrophobicity [7]. In the presence of CMCO-A, -B and commercial additive, the surface hydrophobicity of MP was lower than that of the control group, suggesting their cryoprotective effects on protein structures. CMCO, an ampholytic saccharide with strong polarity, may interact with proteins and increase their hydrophilicities. Consequently, undesirable protein aggregation was inhibited as evidenced by the reduced hydrophobic interaction [38].

To further elaborate the storage stability of MPs, the contents of their sulfhydryl groups were measured in the presence of different cryoprotectants (Figure 2b). During the 60 days of storage, the sulfhydryl content of the control group decreased significantly from 6.22 to 4.08 mol/10^5^ g, while the addition of CMCO-A and B mitigated such decrease with the final sulfhydryl contents of 4.31 mol/10^5^ g and 4.73 mol/10^5^ g, respectively. Moreover, the CMCO-B group can achieve similar sulfhydryl contents of MP to the commercial group (4.69 mol/10^5^ g). The sulfhydryl group is chemically reactive. It could be easily oxidized to form disulfide bonds under long-term frozen storage [33], leading to serious protein aggregation, denaturation, and deterioration of food quality [39]. Owing to the strong reducing power of oligosaccharide, CMCO could inhibit the oxidation and conversion of SH to disulfide bond, thus maintaining the storage stability of MP [14].

#### 3.1.4. Intrinsic Fluorescence Intensity

**Figure 3:**
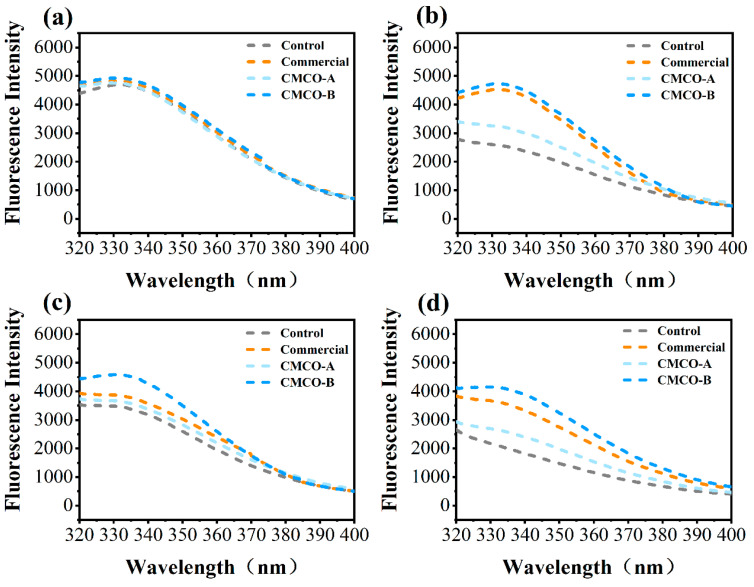
Effects of CMCO on fluorescence intensity of MP at different storage time: (**a**) day 0, (**b**) day 15, (**c**) day 30, and (**d**) day 60.

The intrinsic fluorescence intensity (FI) of MP in frozen surimi was characterized by using fluorescence spectroscopy. It reflected the changes of chemical environment of tryptophan (Trp) residues, which suggested the variation of protein tertiary structures [40]. As shown in Figure 3, the FI of all MP samples was similar at Day 0, and decreased as far as the storage period elapsed, indicating the exposure of Trp residue towards solvent [41]. These results agreed with findings about the structural integrity of MP molecules (Ca^2+^-ATPase activity in Figure 1b), which also experienced significant decreases as the tertiary structure of MP became collapsed during long-term frozen storage [41]. In the presence of different cryoprotectants, reduction of FI became less obvious compared to the control group, and relatively higher FI was observed for the CMCO-B group than the CMCO-A during the entire storage period. A similar charge-dependent cryoprotective behavior of ampholetic saccharides was also observed in our recent study [20], in which CMCh could stabilize the microstructures of wheat gluten in frozen dough, and the protective effect was more obvious as DS of CMCh increased.

### 3.2. Effect of CMCO on Gel Behaviors of Frozen Surimi

#### 3.2.1. Effect of CMCO on the Microstructure of Gels Prepared from Frozen Surimi

**Figure 4:**
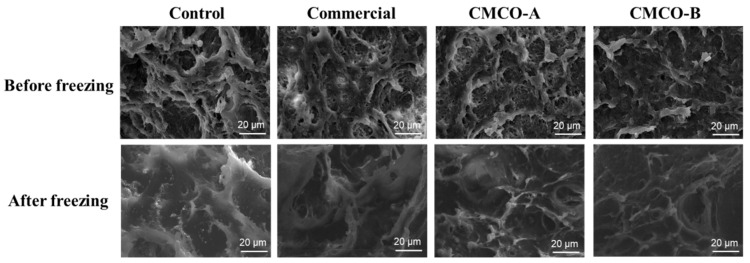
Microstructure of the gels prepared from frozen surimi with different cryoprotectants before and after storage for 60 days.

A porous protein network is formed during the two-stage thermal processing of surimi, which determines many important quality attributes of the gel products, such as texture, shelf life and digestion etc. [42,43]. Therefore, SEM was applied to observe the microstructure of gels prepared from frozen surimi. As shown in Figure 4, all gel samples of fresh surimi exhibited reticular and continuous protein network. After frozen storage, the gel matrix became loose for the control group, seen from the increase of heterogenic pore sizes. In contrast, the addition of cryoprotectants alleviated the breakdown of honeycomb-like network of surimi gels, which were beneficial to the mechanical strength of gel matrix. Besides, a more continuous and ordered protein architecture was observed for surimi gel of the CMCO-B group than that of the CMCO-A. A similar phenomenon was also reported by Tan et al. [13], who investigated the gel morphology of frozen surimi treated by cellulosic oligosaccharide.

#### 3.2.2. Effect of CMCO on the Rheological Properties of Surimi Gels

**Figure 5:**
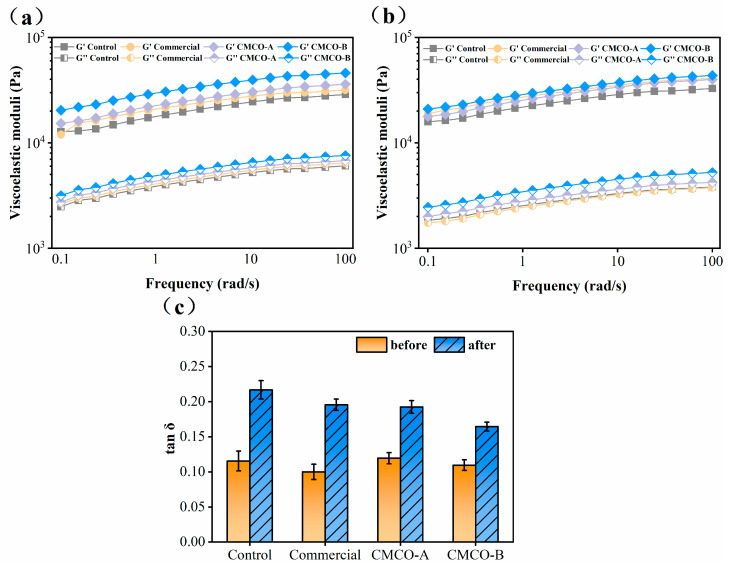
Dynamic rheological properties of the gels prepared from frozen surimi before (**a**) and after (**b**) frozen storage, and (**c**) tan δ of surimi gel within the linear viscoelastic region. Values with different uppercase letters indicate statistically significant difference among samples with different cryoprotectants at the same storage time (*p* < 0.05). Values with different lowercase letters indicate significant difference among samples with the same cryoprotectants at different storage time (*p* < 0.05).

The rheological properties of surimi gels also changed significantly as the MP became denatured during frozen storage. As shown in Figure 5a,b, the elastic moduli (G’) of all surimi gels were higher than those of the viscous moduli (G”) at both day 0 and day 60, indicating their solidlike behaviors. To better illustrate the gel characteristics, loss tangents (tan δ) of surimi gels during frequency sweep were recorded (Figure 5c). The reduced elasticities were observed for all groups of surimi gels, as evidenced by their ever-increased tan δ (i.e., less elastic) after freezing storage. These results were correlated with the formed heterogeneous gel networks prepared from the stored surimi (Figure 4). In the cases of cryoprotected surimi, their gel structures were maintained, thus exhibiting lower tan δ compared to the control. After 60 days, the lowest tan δ was obtained for the CMCO-B group at 0.16. These observations were in consistent with the effect of antifreeze proteins on rheological properties of the frozen surimi [33,42].

#### 3.2.3. Effect of CMCO on the Gel Strength and Water-Holding Capacity

**Figure 6:**
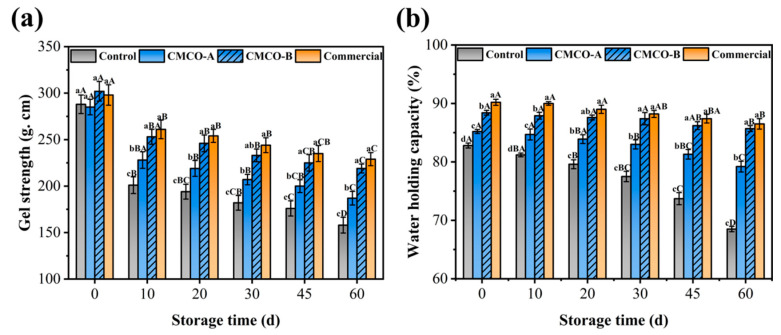
Effects of CMCO on the mechanical strength (**a**) and water-holding capacity (**b**) of the gels prepared from frozen surimi at different storage time. Values with different uppercase letters indicate statistically significant difference among samples with different cryoprotectants at the same storage time (*p* < 0.05). Values with different lowercase letters indicate significant difference among samples with the same cryoprotectants at different storage time (*p* < 0.05).

Gel weakening is one of the most typical quality deteriorations for surimi products, which occurs during the whole process of cold chain transportation, storage and communications [44,45]. Thus, the gel strengths of frozen surimi with different cryoprotectants were characterized (Figure 6a). Few significant differences were observed among the samples at day 0, and the weakening effect was observed as storage time prolonged. After 60 days, gel strength of the control group exhibited the most significant decrease from 288 to 158 g·cm. The weakening effect was suppressed in the presence of cryoprotectants, and the highest gel strength of 219 g·cm was obtained for the CMCO-B group. As previously discussed (Section 3.1), introduction of CMCO to frozen surimi may preserve the MP integrity (solubility, Ca^2+^-ATPase, sulphydryl, and intrinsic FI), which stabilized protein networks and enhanced mechanical strengths of surimi gels.

In addition to the gel strength, WHC is another important parameter to evaluate edible qualities of gel-type foods. As shown in Figure 6b, WHC of all surimi gels kept decreasing during storage, which could be owing to the muscle filament contraction and protein denaturation/ tertiary structural changes [5]. In the presence of different cryoprotectants, such reduction of WHC became less obvious. After 60 days, the CMCO-A, CMCO-B, and commercial groups exhibited significantly higher WHC (79.20%, 85.70%, and 86.50%, respectively) than the control group (68.50%). The improved WHC may be due to the cryoprotective effect of CMCO to the gel structures. The intrinsic ampholytic characteristics of CMCO may endow the saccharide superior hydrophilicity, which helped reduce the ice crystallization, improve the gelling property and finally enhance the WHC [12]. In consistent with the results of mechanical strength, as the DS of CMCO increased, the WHC of surimi gels were enhanced (CMCO-B > CMCO-A).

**Figure 7:**
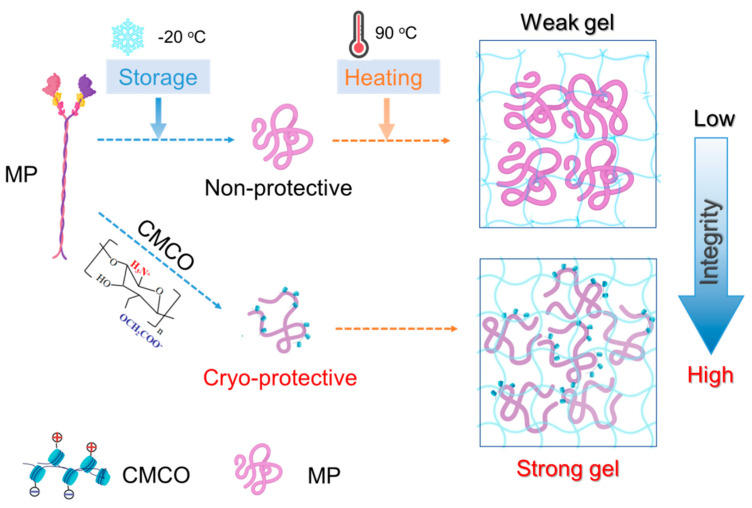
Schematic representation of the cryoprotective mechanism of CMCO for MP in frozen surimi.

Collectively, the cryoprotective mechanism of CMCO can be described from molecular perspective (Figure 7). CMCO could interact with MP through both hydrogen bonding and electrostatic complexation, which replaces the water molecules at MP surface, and prevents the freezing-induced protein aggregation [41,42]. In addition, the ampholytic structure of oligosaccharide could entrap the water, modulate the growth of ice crystals, and stabilize MP structures [46]. Thus, after thermal processing, the gel of cryoprotected surimi exhibited more organized microstructures and improved mechanical properties. It is also noteworthy that the inhibitory effect of saccharide to ice crystallization may also account for the cryoprotective behaviors of CMCO to frozen surimi [47,48,49], and in most cases, both the protein-stabilization and ice-inhibition effects worked concurrently.

### 3.3. Effect of CMCO on the Whiteness and Sensory Quality of Frozen Surimi

**Figure 8:**
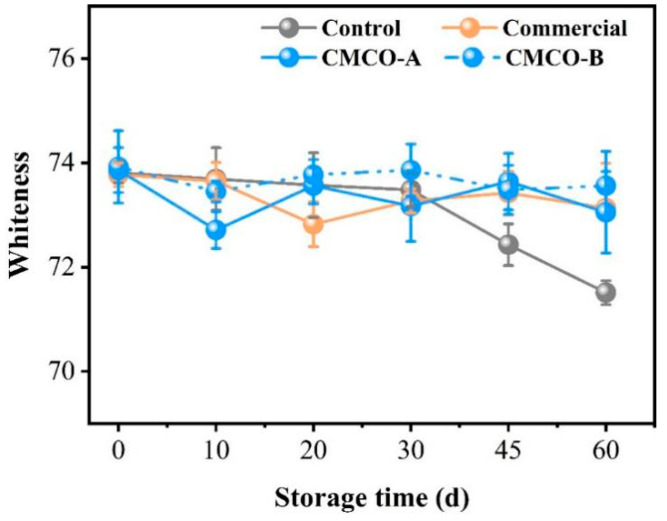
Effects of the CMCO on whiteness of the gels prepared from frozen surimi at different storage time.

Decreased whiteness of gel products made from frozen surimi is a common problem impairing their appearance and edible qualities. As depicted in Figure 8, the whiteness value of the control group kept decreasing from 0 to 60 days. As expected, the addition of cryoprotectants could limit the loss of whiteness of surimi gels, whose values of CMCO-A, -B, and commercial group experienced no significant difference during the entire storage period. Similar phenomenon was also reported by Tao and Walayat [5,8] who attributed the decline of gel whiteness to the combined effects of nonenzymatic protein oxidation and crystallization-induced protein denaturation. The addition of oligosaccharides could prevent those protein deteriorations and thus maintain the whiteness of gels prepared from frozen surimi [50].

Sensory evaluations of all surimi gels were performed by panelists (Table 1). The average sensory scores of surimi gels enhanced in terms of flavor, taste, juiciness, texture, and color through the addition of cryoprotectants. Moreover, gels of the CMCO-B group exhibited slightly higher overall acceptability than that of the CMCO-A and commercial group. In agreement with the results of gel strength, and whiteness (Figure 6), the CMCO-B could act as a high-performance cryoprotectant to improve the edible quality of the frozen stored surimi.

## 4. Conclusions

The present study designated the cryoprotective effects of ampholetic oligosaccharide (CMCO) on the storage stability of frozen surimi. An addition of 0.6% (*w*/*w*) CMCO can alleviate the denaturation of MP in the frozen surimi during 60 days of storage at −18 °C as indicated by their increased salt-protein solubility, Ca^2+^-ATPase activity, and sulfhydryl content. The CMCO-protected MP experienced less conformation changes (reflected by the surface hydrophobicity and FI) than the nonprotected control. Accordingly, the obtained surimi gels demonstrated significantly improved elasticity, mechanical strength, and WHC, and had more uniform microstructure upon thermal processing. Moreover, the cryoprotective effect of CMCO-B (DS: 1.2) was more pronounced than that of CMCO-A (DS of 0.8). The gel strength and WHC of the CMCO-B group were comparable to those of the commercial counterpart (4% sucrose and 4% sorbitol). Results of sensory evaluation showed that CMCO addition also improved the taste, smell, texture, juiciness, whiteness, and overall acceptability of gels prepared from frozen surimi. Findings from this study deepen the scientific insights of ampholytic saccharides as high-performance cryoprotectants in food industry.

## Figures and Tables

**Table 1:** Effect of CMCO on sensory attributes of the gels prepared from frozen surimi after 60 days of storage.

	Control	CMCO-A	CMCO-B	Commercial
Taste	3.78 ± 0.43 ^b^	4.18 ± 0.31 ^a^	4.34 ± 0.35 ^a^	4.22 ± 0.27 ^ab^
Smell	4.09 ± 0.45 ^a^	4.38 ± 0.35 ^a^	4.51 ± 0.38 ^a^	4.46 ± 0.40 ^a^
Texture	3.60 ± 0.25 ^b^	3.93 ± 0.34 ^ab^	4.17 ± 0.27 ^a^	4.20 ± 0.32 ^a^
Juiciness	3.52 ± 0.28 ^b^	4.38 ± 0.31 ^a^	4.51 ± 0.35 ^a^	4.46 ± 0.30 ^a^
Color	4.08 ± 0.24 ^a^	4.12 ± 0.22 ^a^	4.10 ± 0.25 ^a^	4.15 ± 0.33 ^a^
Overall acceptability	3.93 ± 0.35 ^b^	4.38 ± 0.27 ^ab^	4.67 ± 0.34 ^a^	4.49 ± 0.31 ^a^

Different letters indicate statistically significant differences among samples with different cryoprotectants (*p* < 0.05).

## Data Availability

Not applicable.

## Supplementary Materials

The following supporting information can be downloaded at: https://www.mdpi.com/article/10.3390/foods11030356/s1, Figure S1: The chemical structure of carboxymethyl chitosan; Figure S2: A schematic demonstration to the processing of surimi.
