# Supplementary material for: Cryoprotective Roles of Carboxymethyl Chitosan during the Frozen Storage of Surimi: Protein Structures, Gel Behaviors and Edible Qualities
Source: Foods. 2022 Jan 26;11(3):356. doi: 10.3390/foods11030356 (PMC8833919; doi:10.3390/foods11030356)
Supplement: Supplementary file 1 [file foods-11-00356-s001.zip › foods-1547825-supplementary.pdf]

Supporting Information

Cryo-protective roles of carboxymethyl chitosan during the frozen storage of surimi: protein structures, gel behaviors and edible qualities

Xiangwei Zhu [1,*], Minglang Zhu [1], Diheng He [1,] Xueyin Li [1], Liu Shi [2], Lan Wang [2], Jianteng Xu [3], Yi Zheng [3], Tao Yin [4*],

[1] National "111" Center for Cellular Regulation and Molecular Pharmaceutics, Key Laboratory of Fermentation Engineering (Ministry of Education), Hubei Key Laboratory of Industrial Microbiology, Hubei University of Technology, Wuhan, 430068, China.; xiangwei@ksu.edu (X. Z.); datouzhu1216@163.com (M. Z.); hdiheng@163.com (D. H.); xiali12282021@163.com (X. L.)

[2] Institute for Farm Products Processing and Nuclear-Agricultural Technology; Hubei Academy of Agricultural Science; Wuhan, 430064, China; shiliu_hzau@163.com (L. S.); lilywang_2016@163.com (L. W.)

[3] Department of Grain Science and Industry, Kansas State University, Manhattan, KS 66506, United States; jianteng@ksu.edu (J. X.); yzheng@ksu.edu (Y. Z.)

[4] College of Food Science and Technology, Huazhong Agricultural University, Wuhan 430070, China; yintao@mail.hzau.edu.cn (T. Y.)

[*] Correspondence: xiangwei@ksu.edu (X. Z.); yintao@mail.hzau.edu.cn (T. Y.)

Tel.: +86-182-7189-3897 (X. Z.)

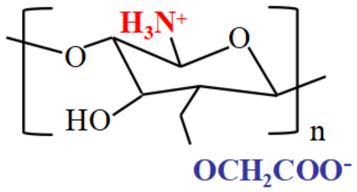

Figure S1. The chemical structure of carboxymethyl chitosan.

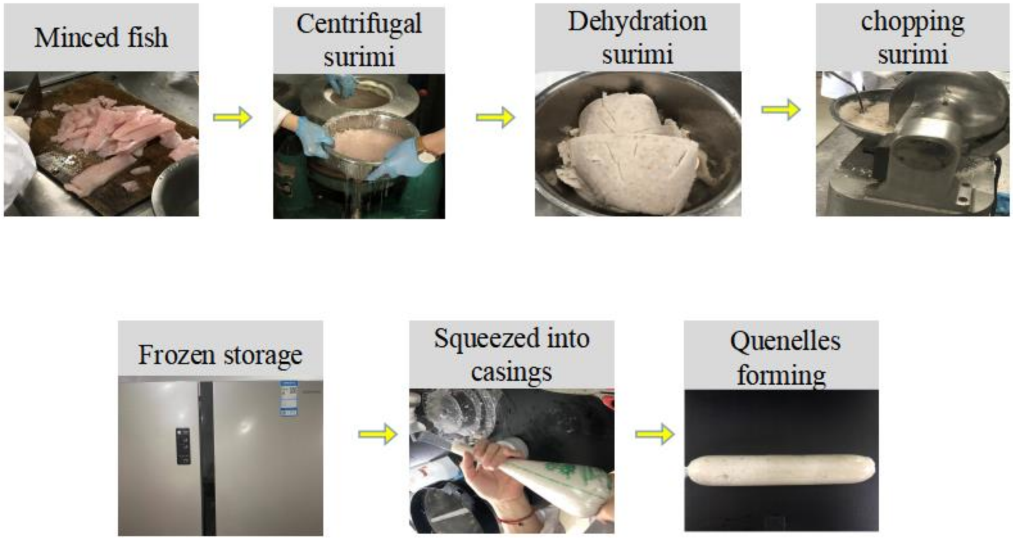

Figure S2. A schematic demonstration to the processing of surimi.
